# Combining Photodegradation in a Liquid-Core-Waveguide Cell with Multiple-Heart-Cut Two-Dimensional Liquid Chromatography

**DOI:** 10.1021/acs.analchem.2c01928

**Published:** 2022-07-29

**Authors:** Mimi J. den Uijl, Yorn J.H.L. van der Wijst, Iris Groeneveld, Peter J. Schoenmakers, Bob W. J. Pirok, Maarten R. van Bommel

**Affiliations:** † van ’t Hoff Institute for Molecular Sciences, Analytical-Chemistry Group, University of Amsterdam, Science Park 904, 1098 XH Amsterdam, The Netherlands; ‡ Centre for Analytical Sciences Amsterdam (CASA), Amsterdam, The Netherlands; § Amsterdam Institute for Molecular and Life Sciences, Division of Bioanalytical Chemistry, Vrije Universiteit Amsterdam, De Boelelaan 1108, 1081HZ Amsterdam, The Netherlands; ∥ Amsterdam School for Heritage, Memory and Material Culture, Conservation and Restoration of Cultural Heritage, University of Amsterdam, P.O. Box 94552, 1090 GN, Amsterdam, The Netherlands

## Abstract

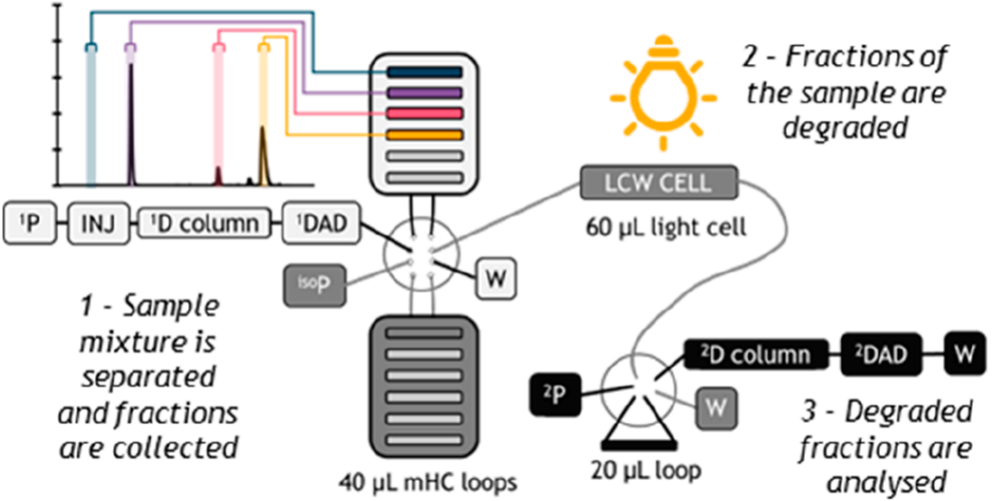

Photodegradation greatly affects everyday life. It poses challenges when food deteriorates or when objects of cultural heritage fade, but it can also create opportunities applied in advanced oxidation processes in water purification. Studying photodegradation, however, can be difficult because of the time needed for degradation, the inaccessibility of pure compounds, and the need to handle samples manually. A novel light-exposure cell, based on liquid-core-waveguide (LCW) technology, was embedded in a multiple-heart-cut two-dimensional liquid chromatography system by coupling the LCW cell to the multiple-heart-cut valve. The sample was flushed from the heart-cut loops into the cell by an isocratic pump. Samples were then irradiated using different time intervals and subsequently transferred by the same isocratic pump to a second-dimension sample loop. The mixture containing the transformation products was then subjected to the second-dimension separation. In the current setup, about 30–40% of the selected fraction was transferred. Multiple degradation products could be monitored. Degradation was found to be faster when a smaller sample amount was introduced (0.3 μg as compared to 1.5 μg). The system was tested with three applications, that is, fuchsin, a 19th-century synthetic organic colorant, annatto, a lipophilic food dye, and vitamin B complex.

## 1 Introduction

Photodegradation is the process in which molecules undergo a change due to UV/vis irradiation. It poses challenges in many different fields, but also some opportunities. Food products, especially those stored over time, can be susceptible to light, which affects their shelf-life time. For other products pulsed-light processing is used as a disinfection technique.^1−4^ Light may cause healthy food ingredients, for example, vitamins, to degrade and thus lose their nutritional value.^5^ The problem becomes even larger when food ingredients are transformed into products that can be harmful.^6^ Understanding photodegradation and its pathways can help prevent food spoilage and aid in the development of packaging materials that reduce light-induced transformation. Another field in which light degradation poses serious problems is cultural heritage. Organic colorants applied as dyes degrade over time, resulting in art objects, such as textiles, furniture, drawings, and paintings, changing color and losing their historical value.^7−11^ Knowledge about the degradation pathways and the influence of other parameters on light degradation can help us preserve valuable objects for future generations and understand the original appearance of objects of art.

Conversely, there are fields that exploit photodegradation advantageously. For example, in water-purification systems, advanced-oxidation processes are applied to reduce the concentration of organic matter.^12,13^ In such processes, hydrogen peroxide can be used in combination with UV light to eradicate toxic compounds in drinking water. The photodegradation reactions occurring in such systems (i.e., radical reactions) require further study, since the byproducts formed could possibly be harmful.^14^

To study photodegradation, several methods exist.^7,15−17^ The simplest method is to illuminate a solution of a single dissolved compounds with a lamp. Unfortunately, degradation studies often report exclusively on the degradation of the starting compound, rather than studying the pathways and concentrations of all different degradation products.^5,18^ Introducing more compounds and parameters inevitably leads to a tangle of different degradation pathways that occur simultaneously. To establish unambiguous links between a compound and its degradation products, it is preferable to isolate it prior to degradation, so as to circumvent tedious (or even impossible) measurements and data analysis. Unfortunately, few compounds are available in pure form or are very difficult and expensive to obtain. To determine the reaction rates, different time points should be recorded, so sufficient material must be available.

Photodegradation techniques generally require time and manual handling (e.g., degradation during a week).^7^ Often a subsequent manual extraction is needed, introducing more sources of error. If more compounds and more parameters (e.g., time, catalysts, oxygen supply) are studied, the experimental procedure becomes very cumbersome and lengthy.

An alternative method to perform photodegradation research more efficiently was recently published.^7,19,20^ In this study a liquid-core-waveguide (LCW) photodegradation cell was developed. This LCW cell comprises of an amorphous-Teflon (AF2400) tubing, which has a lower refractive index (RI = 1.29) than typical solvents. When the LCW cell is filled, the light is guided through the cell by total internal reflection, irradiating the sample inside.^21^ However, just as with conventional techniques, this approach focused on solutions of pure compounds without prior liquid chromatography (LC) separation and the accompanying dilution.^7,19^ Investigating mixtures is feasible with this system, however, it is complicated, since the link between parent molecules and their degradation products will be difficult. One way to reduce the complexity of the sample undergoing degradation is to separate the compound of interest from the mixture with LC separation. If the reaction products are to be characterized with the aid of LC, the resulting setup would be akin to a two-dimensional liquid chromatography (2D-LC) system with a light cell implemented as the modulator.^22^ Photodegradation is then a specific form of reaction modulation or transformative modulation.^23−25^ To our knowledge, such a 2D-LC system with light modulation has never been realized before. Normally, in 2D-LC, a high orthogonality should be achieved between the two different (first-dimension, ^1^D, and second-dimension, ^2^D) separation systems.^26^ However, in reaction modulation, the situation might be opposite. The difference between the two separations is due to the sample being transformed, and the separation systems do not need to be different.

In this work an online photodegradation system was developed that allows a fast and efficient study of solutions of a number of individual compounds from a complex mixture. This approach is based on a (LCW) reactor embedded in a multiple-heart-cut 2D-LC with reversed-phase liquid chromatography (RPLC) in the ^1^D and ^2^D separations. The aim was to investigate the effects of a number of 2D-LC method parameters and the concentration of compounds on the design and application of the LCW reactor and to evaluate its performance by comparing the products obtained at varying residence times with control samples. Finally, it was aspired to demonstrate the versatility of the new approach by applying it to examples from cultural heritage and food ingredients. These include fuchsin,^27−29^ which is a mixture of organic colorants that was extensively used in the late 1900s as dye for textile, food, and wine, but is now used as a biological staining agent, annatto-seeds extract,^30,31^ used widely in the food industry to color hydrophobic products, such as cheese and butter, and a vitamin-B-complex formulation,^32^ a mixture of essential vitamins.

## 2 Materials and Methods

### 2.1 Chemicals

HPLC-quality water was obtained from a purification system (*R* = 18.2 MΩ cm; Arium 611UV, Sartorius, Göttingen, Germany). Methanol (MeOH, LC-MS grade) was obtained from Biosolve (Valkenswaard, The Netherlands). Crystal violet (CV; ≥90%), eosin Y (EY; 99%), riboflavin (RF; ≥98%), ammonium formate (AF; ≥99.0%), and formic acid (FA; ≥95%) were purchased from Sigma-Aldrich (Zwijndrecht, The Netherlands).

Fuchsin was a gift from the Cultural Heritage Agency of The Netherlands (RCE, Amsterdam, The Netherlands). Annatto seeds were purchased from De Peperbol (Amsterdam, The Netherlands), and the Davitamon vitamin-B pills were purchased from Etos (RF concentration 2.8 mg per pill, Amsterdam, The Netherlands).

### 2.2 Instrumentation

#### 2.2.1 LC-LCW-LC-DAD

An Agilent 1290 Infinity 2D-LC system (Agilent, Waldbronn, Germany) was used for all experiments in this study. The system was comprised of two binary pumps (G4220A), two diode-array detectors (DAD, G4212A) equipped with Agilent Max-Light Cartridge Cells (G4212–6008, *V*_0_ = 1.0 μL), and a G4226A autosampler as injector. A 2-position 10-port valve configured as an 8-port valve (G4243A) was used with two multiple-heart-cut (mHC) valves (G64242–64000) with 40 μL loops installed. Two RPLC Zorbax Eclipse Plus C18 columns (Agilent) were used, with dimensions of 2.1 × 150 mm (3.5 μm particle size) and 4.6 × 50 mm (1.8 μm) in the first- and second-dimension, respectively. An 1100 Agilent isocratic pump (G1310A) was used to transfer the sample from the mHC valve to the LCW cell and eventually to the loop.

The LCW cell (i.d. 800 μm, o.d. 1000 μm, length 120 mm, volume 60 μL, pressure limit 0.5 MPa) was placed in a light box created by DaVinci Laboratory Solutions (Rotterdam, The Netherlands), which is described and validated in previous research^19,20^ and shown in Supporting Information, Section S-1. The light source for degradation was a cold-white (400–700 nm) LED lamp (MCWHF2, Thorlabs, Newton, NJ, United States), which was coupled to a light fiber cable (M113L01, Thorlabs) with a 400 μm core diameter, a UV/vis collimator from Avantes (COL-UV/vis, Apeldoorn, The Netherlands) and a plano-convex lens (*f* = 35 mm, Thorlabs, LA4052-ML) to couple the light from the source into the LCW cell. The LED was controlled by a LED driver (LEDD1B, Thorlabs). The lightbox was also equipped with a 6-port switching valve to transfer the sample from the light box to the ^2^D separation. The 6-port switching valve was equipped with a loop of 20 μL.

### 2.3 Methods

#### 2.3.1 Analytical Methods

**Figure 1:**
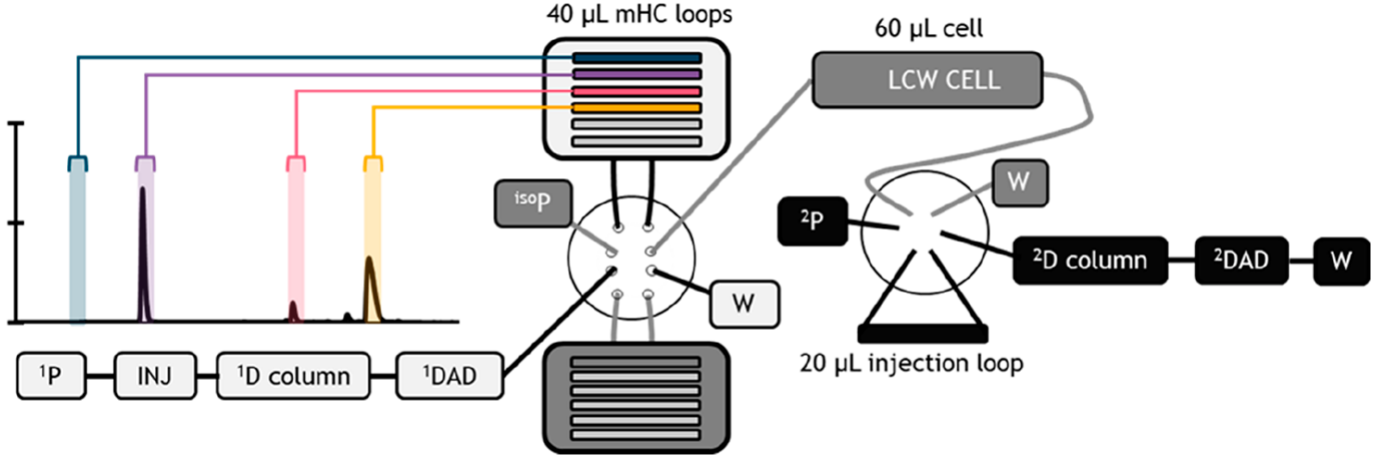
Schematic illustration of liquid chromatography–liquid-core-waveguide cell–liquid chromatography setup. The three different flow paths are indicated with a different shade of gray (white, gray, and black). On the top left, the ^1^D chromatogram of the test mix is shown with the peak cuts for the blank, riboflavin, crystal violet, and eosin Y (semitransparent rectangles).

In this work a multiple-heart-cut two-dimensional liquid-chromatography setup is developed and expanded to allow photodegradation in a LCW cell between the two separation dimensions. A binary pump, autosampler, ^1^D column, the ^1^D DAD, and 10-port valve were connected in series. This latter valve was equipped with two multiple-heart-cut (mHC) valves. An isocratic pump was used to transfer the ^1^D fractions stored in the loops of the mHC valves to the LCW cell. This latter cell was coupled to a 6-port valve, equipped with a 20 μL loop, which connected the ^2^D binary pump to the ^2^D column. A schematic overview of the setup is shown in Figure 1.

For the LC analysis of all samples, mobile-phase components A and B consisted of mixtures of aqueous buffer and MeOH in ratios of 95/5 [v/v] for mobile phase A and 5/95 [v/v] for mobile phase B. The aqueous buffer contained 10 mM ammonium formate at pH = 3, prepared by adding 0.390 g of formic acid and 0.095 g of ammonium formate to 1 L of water. Mobile-phase components A and B were used for both the ^1^D and ^2^D separation. The ^1^D flow rate was set to 0.4 mL/min. The ^1^D gradient program started isocratically at 100% A from 0 to 1 min, followed by a linear gradient to 100% B in 7 min, maintained for 2 min at 100% B, and finally returned to 100% A in 2 min. The composition was kept at 100% A for the remainder of time before starting a new run. The ^2^D flow rate was set to 0.3 mL/min. The ^2^D gradient program started isocratically at 100% A from 0 to 1 min, followed by a linear gradient to 100% B in 7 min, 100% B for 1 min, and back to 100% A in 0.01 min. The mobile phase was kept at 100% A for at least 12 min, depending on the degradation time. The isocratic pump delivered a 50/50 [v/v] mixture of A and B at a flow rate of 0.05 mL/min when in operation.

In the multiple-heart-cut method, a ^2^D gradient stop time of 11.95 min and a cycle time of 12.00 min were used for both the 0 min (10 min residence time, no illumination) and 10 min (10 min residence time with illumination) degradations. In general, the total duration of the ^2^D method was 2 min longer than the residence time. For example, for a 30 min degradation the ^2^D cycle time was 32 min and the gradient stop time 31.95 min. The time-based heart-cut method was used, and the cut times for all compounds studied in this work can be found in te Supporting Information, Section S-2. In all cases, an extra blank cut was taken before the elution of the first peak.

The isocratic pump (^iso^P, Figure 1) was used to transfer the sample from the mHC loop (indicated in Figure 1) to the LCW cell and to transfer the degraded sample from the cell to the ^2^D injection loop (see Figure 1). When the ^2^D method was started, the ^iso^P was operated for 1 min (50 μL) to bring the sample from the mHC loop to the LCW cell. The flow rate of the ^iso^P was then set to zero to degrade the sample until 1.6 min (80 μL) before the start of the next modulation, where the sample was flushed to the ^2^D injection loop. For example, in a method where the sample was degraded for 10 min and the heart-cut of the last peak ended at 8.81 min, the isocratic pump was operated at a flow rate of 0.05 mL/min from 8.81 until 9.81 min, 0 mL/min from 9.82 min until 19.20 min, and 0.05 mL/min from 19.21 until 20.81 min. This 12 min cycle was then repeated for the number of cuts taken.

#### 2.3.2 Sample Preparation

A test mixture was used of riboflavin (RF, 25 ppm), eosin Y (EY, 20 ppm), and crystal violet (CV, 5 ppm) in a H_2_O/MeOH (70/30%) solution. Fuchsin was dissolved in in H_2_O/MeOH (50/50%) with a concentration of 50 ppm.

Five annatto seeds (0.1620 g) were extracted in 5 mL of MeOH/H_2_O (75/25%) and sonicated for 20 min in an ultrasonic bath. After sonicating, the solution was passed through a PTFE filter (0.45 μm).^33^

Two pills of vitamin B (0.8820 g) were grinded, dissolved in 20 mL of MeOH, and sonicated in an ultrasonic bath for 20 min. After this, the liquid was passed through a PTFE filter (0.45 μm) and then diluted in a vial to reach a H_2_O/MeOH ratio of 50/50%.^34^

### 2.4 Data Processing

The chromatograms were processed with Agilent OpenLAB CDS software (Agilent, Santa Clara, CA, U.S.A.). Calculations and figures were performed with MATLAB R2018a (Mathworks, Woodshole, MA, U.S.A.) and Microsoft Excel.

## 3 Results and Discussion

In this research, an online system was developed that allowed fast and efficient study of the in-solution photodegradation of a number of individual compounds obtained from the separation of a mixture. The system was based on a liquid-core-waveguide (LCW) reactor embedded in a multiple-heart-cut 2D-LC (mHC-2D-LC) setup. In this section, the setup and optimizable parameters will first be discussed (Section 3.1). The degradation efficiency will be evaluated (Section 3.2). To demonstrate the potential of the system three applications will be described (Sections 3.3–3.5).

### 3.1 Design of the System

To facilitate quick and efficient light degradation, an LCW cell was incorporated in a mHC-2D-LC setup. This LCW cell was previously coupled to an LC-DAD for studying degradation products.^7,19^ In those studies, the sample was directly introduced into the light cell either manually^7,19^ or by automated sampling with a multipurpose sampler.^20^ In the setup used in the present research, however, the sample was automatically transferred to the LCW from the ^1^D separation (Figure 1). In the so-called time-based-sampling method, the time frame of the eluting compounds must be inserted in the method. Therefore, the retention times of the targeted analytes in the ^1^D system had to be determined first. The cut times for all compounds studied in this work can be found in Supporting Information, Section S-2. After the cut times were established, a sample was injected by an autosampler into the ^1^D column. The components of interest were transferred to loops in the mHC valve. In this work the number of peaks collected did not exceed the capacity of a single “deck” (multiloop valve) and the first ^2^D method only started after the last fraction was stored.

When the mHC is used to store the separated components prior to exposure in the LCW, an additional loop is required to transfer the last exposed component to the ^2^D separation. Normally in mHC-2D-LC, the ^2^D pump is directly connected to the heart-cut valve. In the present setup an additional pump (^iso^P in Figure 1) was inserted to transfer the component of interest from the mHC valve to the LCW cell. The outlet of the LCW was connected to a six-port valve that served as modulation valve and injection valve for the ^2^D system. The storage loops were eluted one by one to the LCW cell, where they were either stored or degraded for a selected time interval. The exact timing had to be adapted, depending on the degradation time. When the sample was injected to the ^2^D separation, the next component in a storage loop was transferred to the LCW and the cycle repeats. To ensure the start of the last ^2^D separation, an extra ^1^D cut was required, since the presence of the additional flow path and the light cell could not be detected by the software. In the present setup, a blank ^2^D run was performed before the components of interest were transferred to the second dimension, since all stored analyte peaks were first transferred to the LCW cell before being transported to the ^2^D system. This extra cut was always taken in the method between 5 and 5.1 min. The timings established for the different degradation times are listed in Supporting Information, Section S-3 and Tables S-2–S-4.

Because the LCW cell has a pressure limit of 0.5 MPa, the flow rates used in the ^1^D and ^2^D LC cannot be used to fill and empty the light cell. This is circumvented by using wider tubing (between the LCW and the six-port valve and for the loop in this valve) and a low operating flow rate for the isocratic pump (see Figure 1). The volume of the mHC storage loops (40 μL) is smaller than that of the LCW cell (60 μL), which in turn is larger than the injection loop of the ^2^D system (20 μL). This will result in an inevitable loss of sample when a peak is transferred from the ^1^D column to the ^2^D system. The highest yields were observed with a flush volume of either 80 or 85 μL (pump operating at 1.6 or 1.7 min at 50 μL/min). The ratio of ^2^D peak area to ^1^D peak area for an 80 μL flush volume was found to be 36% for RF, 42% for CV, and 30% for EY. For an 85 μL flush volume, the ratios were 34% for RF, 37% for CV, and 26% for EY. These ratios are seen to be significantly different for the different compounds. This is due to the limited size of the storage loops in the mHC valve (40 μL, corresponding to an elution window of 0.1 min at a ^1^D flow rate of 400 μL/min). A smaller fraction of the sample is transferred when the ^1^D peak is broader. This can be corrected for, resulting in a reduction of the variation between the different analytes. The corrected ratios for an 80 μL flush volume were found to be 39% for RF, 45% for CV, and 43% for EY. For the 85 μL flush volume, the ratios were found to be 37% for RF, 39% for CV, and 36% for EY. Slightly higher ratios were observed for a transfer volume of 80 μL. For riboflavin the effect of the transfer time is seen to be small, whereas it is larger for the later-eluting compounds.

It may be argued that peak-based cutting (based on a detection threshold) is less prone to variations in chromatographic conditions than time-based cutting. However, in the present setup peak-based cutting was only possible with one specific wavelength. Moreover, a blank (first) cut should be additionally programmed (time-based) and the entire program of the isocratic pump must be anchored on the peak-based cutting times. This was not possible with the present setup.

### 3.2 Feasibility of the System

**Figure 2:**
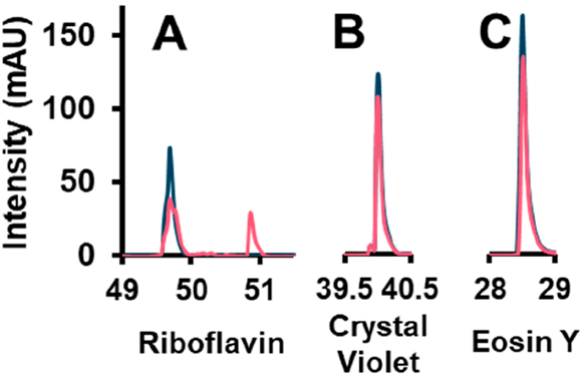
Overlay of chromatograms obtained after 10 min residence time of a fraction cut from the ^1^D effluent in the LCW cell with the light off (blue) and the light on (pink). A, riboflavin (254 nm); B, crystal violet (590 nm); C, eosin Y (520 nm). Note that the *x*-axis is following the ^1^D separation order.

To assess the system, three compounds in a mixture were studied, that is, riboflavin, crystal violet, and eosin Y. The first step in this process was to explore how the analytes behave in the cell, with and without irradiation. The results are shown in Figure 2.

These data were obtained with a modulation time of 12 min, leading to a residence time in the LCW cell of about 10 min. The sample was irradiated with an LED “white” spectrum, including wavelengths ranging from 400 to 800 nm. This illumination source was chosen to mimic indoor conditions without UV light as encountered, for example, in museums or supermarkets. Clear degradation can be seen for riboflavin (Figure 2A). For crystal violet and eosin Y, no degradation products are observed (Figure 2B,C). Some variations are observed in the peak area of the main compounds, but this is likely due to slight variations in the ^1^D retention times between runs, leading to variations in sample transfer. For riboflavin, a number of different degradation products are formed after 10 min. The most intense peak (eluting at 51 min) corresponds to lumichrome, as confirmed by its UV-absorbance spectrum. The complete identification of the degradation products of riboflavin is beyond the scope of this research.

**Figure 3:**
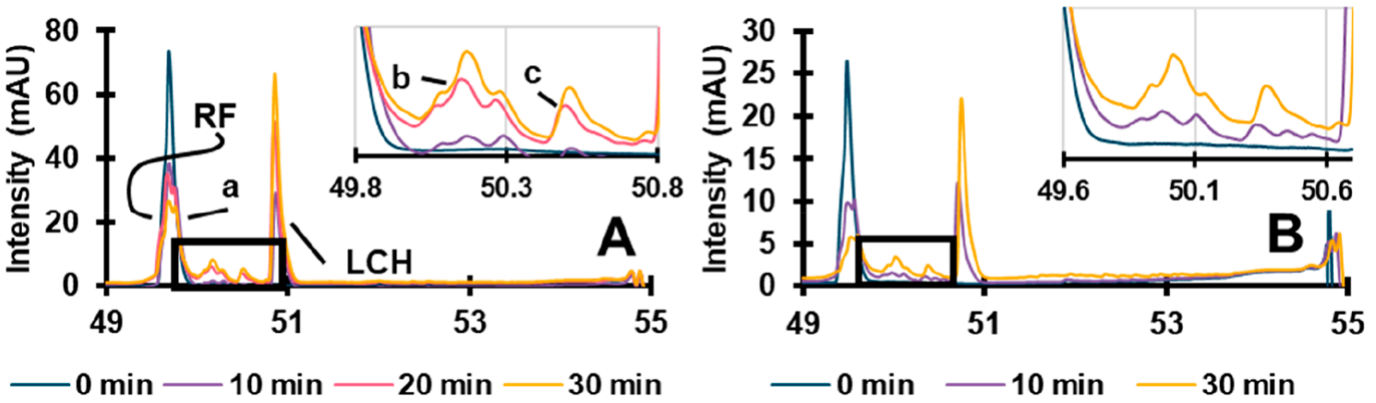
Timeseries of riboflavin (RF) degradation in the LCW cell for ^1^D injection volumes of 15 μL (A) and 3 μL (B): 0 min (10 min with lamp off, blue) 10 min (purple), 20 min (pink, Figure 3A only), 30 min (yellow).

To study the effect of the degradation time, a time series was recorded with irradiation ranging from 0 to 30 min (Figure 3A). Longer residence times in the LCW result in a further degradation. Besides the increase in peak area for the lumichrome (LCH in Figure 3) and other degradation products, a peak arose before the RF peak for longer irradiation times, indicating the formation of a product with a nearly identical ^2^D retention time. For quantitative interpretation (*vide infra*) the two compounds were sufficiently separated and treated independently.

**Figure 4:**
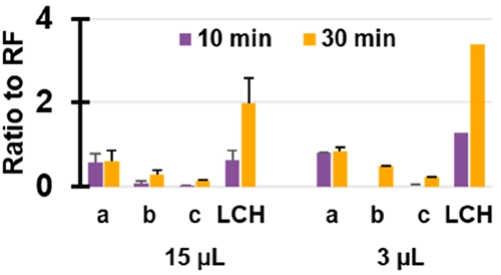
Ratios of peak areas relative to riboflavin for the various degradation products (a, b, c, and lumichrome, LCH) for ^1^D injection volumes of 15 μL (left) and 3 μL (right) for 10- and 30 min irradiation.

The results presented in Figure 3A were obtained by using a ^1^D injection volume of 15 μL, which leads to large sample amounts and, therefore, possible “saturation” of the LCW cell. The intention is to irradiate the collected fraction along the entire length of the cell. However, when saturated, a significant fraction of the light may be absorbed by the sample at the front of the LCW cell, leading to an axial illumination gradient. To test this hypothesis, the ^1^D injection volume of the same sample was reduced by 80% to 3 μL. The results are shown in Figure 3B. While the two sets of overlaid chromatograms in Figure 3 are seemingly similar, the areas of the degradation products are much larger relative to the RF peak in Figure 3B. Clearly, the lower amount of sample introduced into the LCW leads to faster degradation. However, smaller amounts of degradation product are formed, and identification of minor products may be more difficult in the current setup. In Figure 4 the area of the degradation products relative to the total peak area of RF is shown for both the 15 and 3 μL ^1^D injection volumes. Degradation product *a* is formed in the first 10 min, but its relative concentration stays similar thereafter. Hence, its absolute concentration passes through a maximum. The relative peak areas of the other three degradation products all increase over time. Products *b* and *c* were only detected after 30 min degradation at the lower injected concentration. However, for LCH, there is a clear increase visible. The results confirm that a lower analyte concentration results in faster degradation. The injection volume or the absolute amount of sample introduced, should be optimized, so as to avoid saturation of the LCW cell, while exceeding the limits of detection for the degradation products. A more-sensitive detector shifts the optimum toward lower injected amounts.

### 3.3 Application to Fuchsin

**Figure 5:**
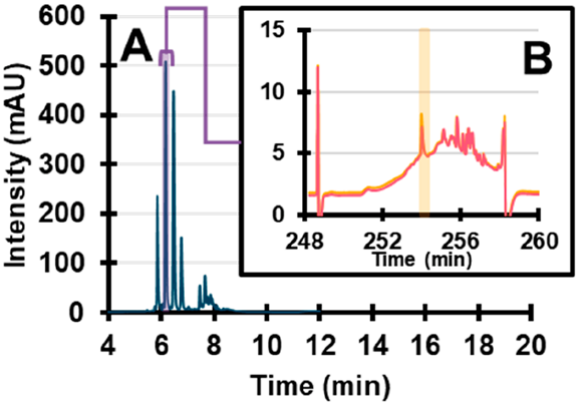
(A) RPLC chromatogram of a fuchsin sample. ^1^D detection wavelength 555 nm. The shaded fraction (M1) is transferred to the LCW and degraded for 4 h. (B) Second-dimension chromatograms of the M1 fraction after 4 h degradation shown in yellow and pink (overlay of two repeat experiments). M1 in ^2^D indicated by the yellow shaded bar. ^2^D detection wavelength 254 nm.

To demonstrate the versatility of the LC-LCW-LC system several different applications were studied. The first of these concerned fuchsin, which is one of the earliest synthetic organic colorants from the 19th century. It was used to dye textile and as a food colorant in the beginning of the 20th century. Nowadays it is used as a biological staining agent for bacteria and sometimes as a disinfectant.^30,31^ The structure of fuchsin is shown in Supporting Information, Section S-4, Figure S-3. It is sold as a mixture of four compounds, which are the triply, doubly, and singly methylated derivatives (known as Magenta III, M3, Magenta II, M2, and Magenta I, M1, respectively) and pararosaniline (M0), which is not methylated (see Figures S-2–S-5). The chromatogram of the mixture (detection wavelength 555 nm) is shown in Figure 5A. The four main compounds can clearly be seen, but impurities are also present. Out of the four main compounds, M0 and M3 are commercially available as pure components, allowing their degradation pathways to be studied individually.^35,36^ In contrast, it has not been possible so far to study the degradation of M1 and M2, which are not available as (more or less) pure compounds. The present LC-LCW-LC setup offers a unique possibility for degradation studies of such individual compounds. Separating the fuchsin mixture prior to compound degradation is essential for this purpose. Figure 5B illustrates the successful degradation of M1.

After 1 h, no degradation products were observed. The present setup readily allows more extensive degradation studies. For that reason, it was chosen to degrade fuchsin (M1) for a longer period. After 4 h, at a wavelength of 254 nm, a number of degradation products were detected (Figure 5B). The present system creates many opportunities to study these products in detail, for example by attaching the setup to a mass spectrometer. The entire experiment (^1^D separation, 4 h degradation, ^2^D separation) was repeated, and the ^2^D chromatograms are overlaid in Figure 5B. Excellent repeatability of the entire process is demonstrated, despite the long degradation times. Slight variations in peak areas (e.g., for M1) can be explained from minor variations in the ^1^D retention times.

### 3.4 Application to Annatto

**Figure 6:**
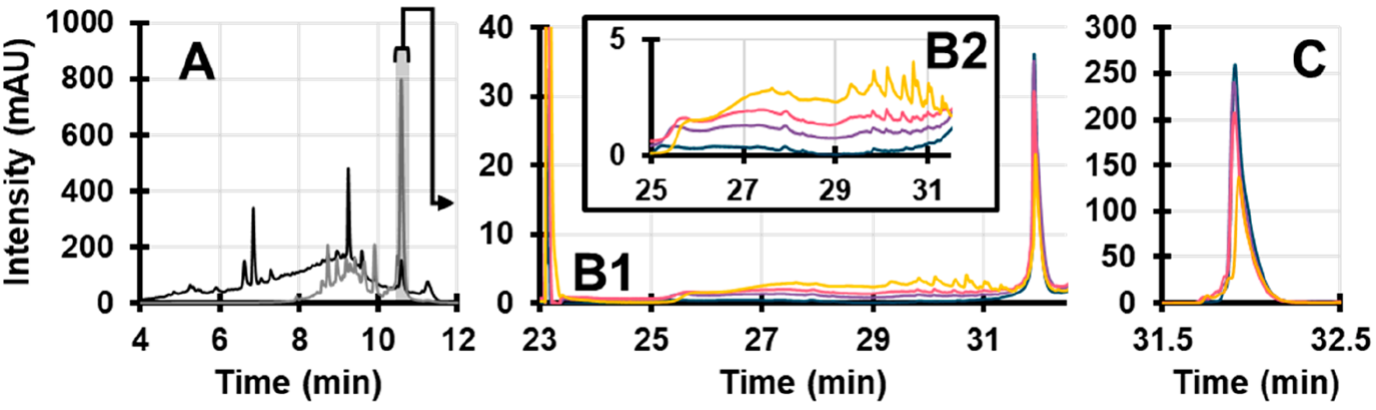
(A) RPLC chromatogram of an annatto seed extract. Detection wavelength 254 nm (black) and 450 nm (gray). The shaded gray fraction is transferred to the LCW and degraded using different time intervals. (B1 and B2) ^2^D chromatograms representing a timeseries of bixin during 0 (blue), 30 (purple), 60 (pink), and 120 min (yellow), recorded at 254 nm. (C) is the same as (B), except recorded at 450 nm.

Annatto is a natural food-coloring agent. It is obtained from the seeds of the Achiote tree and used for coloring many different lipophilic products, such as cheese, ice cream, and margarine. The main coloring compound in the annatto extract is bixin, an apocarotenoid, but there are many other coloring compounds in the extract, as can be seen in the two chromatograms of Figure 6A (recorded at 254 and 450 nm). Studying the photodegradation pathways of bixin can help the food industry prevent food spoilage and improve food packaging. Bixin is one of many compounds present and it is difficult and expensive to obtain it in a purified form. Therefore, degradation studies of bixin have been only been performed on bixin from the annatto extract instead on the pure form.^37,38^

Figure 6B,C show a series of overlaid ^2^D chromatograms of degraded bixin (0, 30, 60, and 120 min), recorded at 254 and 450 nm, respectively. At 450 nm, a small shoulder peak starts to appear before the bixin peak after 30 min exposure. After 60 min, a number of small peaks are visible at 254 nm. This shows the complexity of the degradation of a single compound, and it underlines the value of the current LC-LCW-LC setup. Without the prior separation it would be impossible to deduce which degradation products arise from bixin. This shows how important a preseparation to the photodegradation reaction can be. Again, adding a mass spectrometer to the system will allow detailed interpretation of the structures and pathways.

### 3.5 Application to Vitamin B

**Figure 7:**
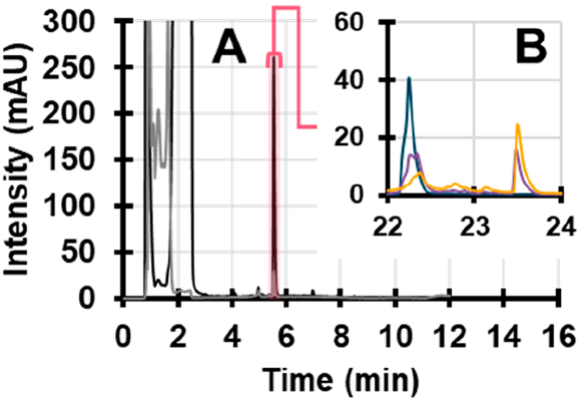
(A) RPLC chromatogram of a vitamin-B-complex formulations, recorded at 254 nm (black) and 300 nm (gray). (B) ^2^D chromatograms corresponding to a timeseries of degradations of RF recorded at 254 nm (0 min, blue, 10 min, purple, 30 min, yellow).

Vitamin B is a group of eight essential vitamins, including thiamine (B_1_), riboflavin (B_2_), niacin (B_3_), pantothenic acid (B_5_), pyridoxine (B_6_), biotin (B_7_), folic acid (B_11_), and cobalamins (B_12_), with many different health benefits. All kinds of vitamin B products are available on the market. As already described in section 3.2, riboflavin is susceptible to light. The riboflavin for the study described in section 3.2 was obtained as a chemical standard which can be studied without prior separation. The LC-LCW-LC setup allows study of riboflavin, and all other compounds present in a vitamin B complex. In Figure 7A, the ^1^D chromatogram is shown for a vitamin-B formulation. Many compounds with vastly different peak areas are observed. The chromatographic behavior of the molecules with many acid groups is poor under these chromatographic conditions.

RF was isolated from the mixture using the ^1^D separation, introduced into the LCW and irradiated using different time intervals. The ^2^D chromatograms after degradation of RF are shown in Figure 7B. This illustrates that RF (or, indeed, any compound) can be studied from complex mixtures, yielding results that are comparable to those obtained with pure standards (see section 3.2 and Figure 3). It should be noted, however, that the conditions in the light cell are different from those experienced in real-world situations. This matrix is acidified and has MeOH present as organic modifier. It is possible that these reaction conditions could lead to different reaction kinetics than present in real-world degradations. It is of importance to compare these degradation studies to the literature present on these subjects. As such, this system should not be considered as a replacement of other aging techniques, but rather as a new tool providing information about degradation mechanisms in a much faster manner. However, understanding the differences in degradation between these matrices proved to be important.^7,8,39^

## 4 Concluding Remarks

In this work, a 2D-LC-based photodegradation workflow was developed to study isolated compounds from mixtures using a liquid-core-waveguide (LCW) cell as reactor. This allowed rapid and efficient studies of components of interest isolated from mixtures. Fractions of the first-dimension effluent were collected by a multiple-heart-cut valve, specifically targeting compounds of interest. A method was developed to transfer the analyte fractions from the first-dimension separation to the light exposure cell and subsequently to the second-dimension separation. About 30% to 40% of the isolated and degraded fraction was transferred. The fastest degradations could be performed with low concentrations of sample (total amount of analyte is approximately 0.3 μg), and care should be taken to avoid saturation of the LCW, which can result in an inhomogeneous irradiation. The system allows us to generate degradation profiles by irradiation of the sample using different time intervals. To our knowledge, this is the first time light-induced reaction modulation has been employed. The method proved to be stable over longer degradation times (up to 4 h). To demonstrate the versatility of the system several samples that contained compounds of interests, as well as many other components, were studied by selecting one of the components present in the mixture. This illustrates the relevance of a preseparation having the advantage that the first separation, the exposure inside the LCW, and the second separation can be carried out online and fully automated. As an example, according to our knowledge, this was the first time a degradation profile of magenta I isolated from fuchsin was demonstrated.

## Supporting Information Available

Additional details about experimental methods, schematic setup of the light box, and structures of fuchsin (PDF)
