# Supplementary material for: Combining Photodegradation in a Liquid-Core-Waveguide Cell with Multiple-Heart-Cut Two-Dimensional Liquid Chromatography
Source: Anal Chem. 2022 Jul 29;94(31):11055–61. doi: 10.1021/acs.analchem.2c01928 (PMC9366730; doi:10.1021/acs.analchem.2c01928)
Supplement: Supplementary file 1 — ac2c01928_si_001.pdf [file ac2c01928_si_001.pdf]

SUPPORTING INFORMATION

Combining photodegradation in a liquid-core waveguide cell with two-dimensional liquid chromatography

Mimi J. den Uijl*[a,b], Yorn J.H.L. van der Wijst[a,b], Iris Groeneveld[b,c], Peter J. Schoenmakers[a,b], Bob W.J. Pirok[a,b], Maarten R. van Bommel[a,b,d]

[a] University of Amsterdam, van 't Hoff Institute for Molecular Sciences, Analytical-Chemistry Group, Science Park 904, 1098 XH Amsterdam, The Netherlands.

[b] Centre for Analytical Sciences Amsterdam (CASA), The Netherlands

[c] Division of Bioanalytical Chemistry, Amsterdam Institute for Molecular and Life Sciences, Vrije Universiteit Amsterdam, De Boelelaan 1108, 1081 HZ Amsterdam, The Netherlands

[d] University of Amsterdam, Amsterdam School for Heritage, Memory and Material Culture, Conservation and Restoration of Cultural Heritage, P.O. Box 94552, 1090 GN, Amsterdam.

(*) Corresponding author

E-mail: M.J.denUijl@uva.nl

Direct line: +31205257040

Contents

S-1. Schematic overview of the LCW-cell box S3

S-2. Optimization of time-based multiple-heart-cut 2DLC method S4

S-3. Time schedule of the multiple-heart-cut two-dimensional liquid chromatography method for different degradation intervals S4

S-4. Structures of fuchsin and its derivatives... S7

S-1. Schematic overview of the LCW-cell box

In Fig. S-1, the LCW-cell box is shown. The sample is introduced at A, where it is transferred to the LCW cell (E). After degradation, the sample is flushed from the cell through PEEK tubing (G) to the ^{2}D-injection loop (I). If the loop is overfilled, the sample is flushed to the waster (K). The ^{2}D binary pump (H) empties the loop onto the ^{2}D column (J). The light comes in through the light fiber cable on the left (B) and moves through the filter wheel (C), which was not operated in this research. In this research, the gas inlet was not controlled (D and F) and the absorption spectra were not collected (right, B).

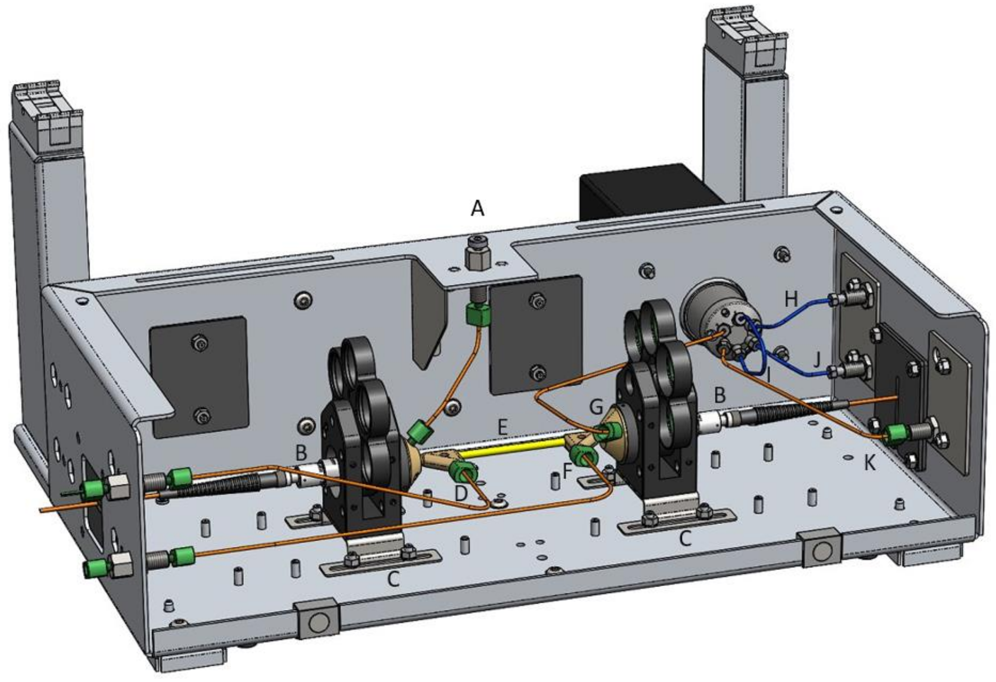

Figure S-1. Schematic overview of the box of the liquid-core-waveguide (LCW) cell. Design and drawings by courtesy of Edwin Beekwilder.

S-2. Optimization of time-based multiple-heart-cut 2DLC method

In Table S-1, the cut times of the four different multiple-heartcut methods are shown.

Table S-1. The start and end times of the cuts of the multiple-heart-cut methods. The last column indicates the method.

Compound	Start time	End time	Method
Fake cut	5.0	5.1	Test mix
Riboflavin	5.2	5.3	Test mix
Crystal Violet	7.69	7.79	Test mix
Eosin Y	8.81	8.91	Test mix
Fake cut	5.00	5.1	Fuchsin
M0	5.83	5.93	Fuchsin
M1	6.14	6.24	Fuchsin
M2	6.44	6.54	Fuchsin
M3	6.73	6.83	Fuchsin
Fake cut	5.00	5.1	Annatto
Bixin	10.61	10.71	Annatto
Fake cut	5.00	5.1	Vitamin B
Riboflavin	5.54	5.64	Vitamin B

S-3. Time schedule of the multiple-heart-cut two-dimensional liquid chromatography method for different degradation intervals

In this research, different methods were used for different samples. In Table S-2-4, these methods are described with their corresponding times and the isocratic-pomp flow rate. In Table S-2, the methods are described for the test mix at different degradation periods. In Table S-3, the methods are described for the different degradation times for fuchsin. In Table S-4, the methods are described for both bixin from the annatto extract and for riboflavin from the vitamin-B extract.

Table S-2. Timings and flow of the multiple-heart-cut 2DLC method for 0-, 10-, 20- and 30-min degradation of the test mix.

Test mix 0 / 10 min	Test mix 20 min	Test mix 30 min	Flow ^{iso}P (mL/min)	Remarks
0.00	0.00	0.00	0.05	Start run
5.00	5.00	5.00	0.05	First (fake) cut
5.20	5.20	5.20	0.05	RF cut
7.69	7.69	7.69	0.05	CV cut
8.70	8.70	8.70	0.05	EY cut
8.81	8.81	8.81	0.05	EY from loop to the LCW
9.81	9.81	9.81	0.05	EY in the LCW

9.82	9.82	9.82	0.00	Stop flow and start degradation EY
19.20	29.20	39.20	0.00	End of degradation EY
19.21	29.21	39.21	0.05	Start EY flow to ^{2}D loop
20.81	30.81	40.81	0.05	EY in the ^{2}D loop + ^{2}D injection valve switch
21.81	31.81	41.81	0.05	CV in the LCW
21.82	31.82	41.82	0.00	Stop flow and start degradation CV
31.20	51.20	71.20	0.00	End of degradation CV
31.21	51.21	71.21	0.05	Start CV flow to ^{2}D loop
32.81	52.81	72.81	0.05	CV in the ^{2}D loop + ^{2}D injection valve switch
33.81	53.81	73.81	0.05	RF in the LCW
33.82	53.82	73.82	0.00	Stop flow and start degradation RF
43.20	73.20	103.20	0.00	End of degradation RF
43.21	73.21	103.21	0.05	Start RF flow to ^{2}D loop
44.81	74.81	104.81	0.05	RF in the ^{2}D loop + ^{2}D injection valve switch
45.81	75.81	105.81	0.05	Fake cut in the LCW
45.82	75.82	105.82	0.00	Stop flow and start degradation fake cut
55.20	95.20	135.20	0.00	Stop degradation fake cut
55.21	95.21	135.21	0.05	Start fake cut flow to ^{2}D loop
56.81	96.81	136.81	0.05	End run

Table S-3. Timings and flow of the multiple-heart-cut 2DLC method for 0- and 60-min degradation of all four fuchsin components (M0, M1, M2, and M3) and for 240 min degradation of M1. The fourth column indicates the remarks about the all-four method, while the fifth column indicates the remarks for 240-min degradation of only fuchsin (magenta I, M1).

**Fuchsin all 0 / 10 min**	**Fuchsin all 60 min**	**Fuchsin only M1 240 min**	**Flow ^{iso}P (mL/min)**	**Remarks fuchsin all**	**Remarks fuchsin only M1**
0.00	0.00	0.00	0.05	Start run	Start run
5.00	5.00	5.00	0.05	Fake cut	Fake cut
5.83	5.83	-	0.05	M0 cut	-
6.14	6.14	6.14	0.05	M1 cut	M1 cut
6.44	6.44	-	0.05	M2 cut	-
6.73	6.73	-	0.05	M3 cut	-
7.73	7.73	7.14	0.05	M3 from loop to the LCW	M1 from loop to the LCW
7.74	7.74	7.15	0.00	Start degradation M3	Start degradation M1
17.12	67.12	246.53	0.00	End of degradation M3	End of degradation M1
17.13	67.13	246.54	0.05	Start M3 flow to ^{2}D loop	Start M1 flow to ^{2}D loop
18.73	68.73	248.14	0.05	M3 in the ^{2}D loop + ^{2}D injection valve switch	M1 in the ^{2}D loop + ^{2}D injection valve switch

19.73	69.73	249.14	0.05	M2 from loop to the LCW	Start flow fake cut to the LCW
19.74	69.74	249.15	0.00	Start degradation M2	Stop flow and start degradation fake cut
29.12	129.12	258.53	0.00	End of degradation M2	Stop degradation fake cut
29.13	129.13	258.54	0.05	Start M2 flow to ^{2}D loop	Start fake cut flow to ^{2}D loop
30.73	130.73	260.14	0.05	M2 in the ^{2}D loop + ^{2}D injection valve switch	End run
31.73	131.73	-	0.05	M1 from loop to the LCW	
31.74	131.74	-	0.00	Start degradation M1	
41.12	191.12	-	0.00	End of degradation M1	
41.13	191.13	-	0.05	Start M1 flow to ^{2}D loop	
42.73	192.73	-	0.05	M1 in the ^{2}D loop + ^{2}D injection valve switch	
43.73	193.73	-	0.05	M0 from loop to the LCW	
43.74	193.74	-	0.00	Start degradation M0	
53.12	253.12	-	0.00	End of degradation M0	
53.13	253.13	-	0.05	Start M0 flow to ^{2}D loop	
54.73	254.73	-	0.05	M0 in the ^{2}D loop + ^{2}D injection valve switch	
55.73	255.73	-	0.05	Fake cut in the LCW	
55.74	255.74	-	0.00	Stop flow and start degradation fake cut	
65.12	315.12	-	0.00	Stop degradation fake cut	
65.13	315.13	-	0.05	Start fake cut flow to ^{2}D loop	
66.73	316.73	-	0.05	End run	

Table S-4. Timings and flow of the multiple-heart-cut 2DLC method for 0-, 10-, 30-, 60- and 120-min degradation of bixin from an annatto extract (columns one to four) and the 0-, 10-, and 30-min degradation of riboflavin from a vitamin-B complex sample (columns five and six).

Annatto 0 / 10 min	***Annatto 30 min***	***Annatto 60 min***	***Annatto 120 min***	***Vit. B 0 / 10 min***	***Vit. B 30 min***	***Flow (mL/min)***	***Remark***
0.00	0.00	0.00	0.00	0.00	0.00	0.05	Start
5.00	5.00	5.00	5.00	5.00	5.00	0.05	Fake cut
10.61	10.61	10.61	10.61	5.54	5.54	0.05	Cut bixin / RF
11.61	11.61	11.61	11.61	6.54	6.54	0.05	Sample to the LCW
11.62	11.62	11.62	11.62	6.55	6.55	0.00	Start degradation sample
21.00	41.00	71.00	131.00	15.93	35.93	0.00	End degradation sample
21.01	41.01	71.01	131.01	15.94	35.94	0.05	Start flow sample to ^{2}D loop
22.61	42.61	72.61	132.61	17.54	37.54	0.05	Sample in the ^{2}D loop + ^{2}D injection valve switch
23.61	43.61	73.61	133.61	18.54	38.54	0.05	Fake cut in the LCW

23.62	43.62	73.62	133.62	18.55	38.55	0.00	Stop flow and start degradation fake cut
33.00	53.00	83.00	143.00	27.93	47.93	0.00	Stop degradation fake cut
33.01	53.01	83.01	143.01	27.94	47.94	0.05	Start fake cut flow to ^{2}D loop
34.61	54.61	84.61	144.61	29.54	49.54	0.05	End run

S-4. Structures of fuchsin and its derivatives

In Figs. S-2-4 the main components of the fuchsin mixture are shown. The structures have a different methylation degree, with pararosaniline (M0, Fig. S-2) being the demethylated form, fuchsin the singly methylated product (M1, Fig. S-3), magenta II having two methylated sites (M2, Fig. S-4) and new fuchsin being the completely methylated form (M3, Fig. S-5).

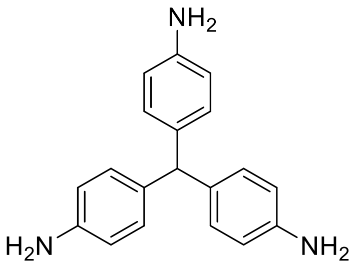

Figure S-2. Structure of pararosaniline or magenta 0 (M0).

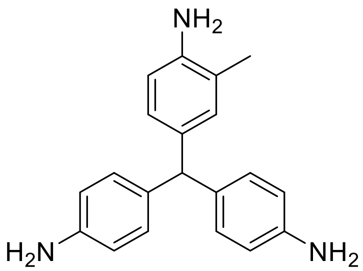

Figure S-3. Structure of fuchsin or magenta I (M1)

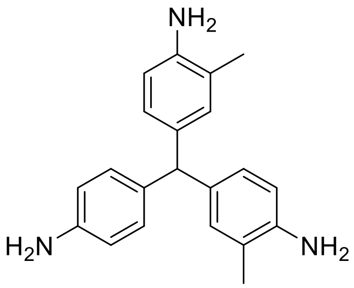

Figure S-4. Structure of magenta II (M2)

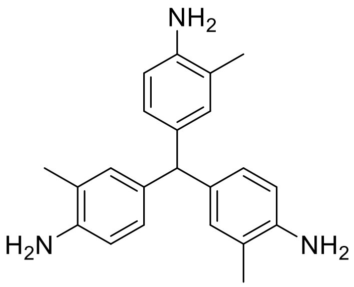

Figure S-5. Structure of new fuchsin or magenta III (M3).
